# Construction of a miRNA-mRNA Network Related to Exosomes in Colon Cancer

**DOI:** 10.1155/2022/2192001

**Published:** 2022-07-05

**Authors:** Wanhui Dong, Dezhen Wu, Sheng Xu, Qingming Sun, Xueping Ci

**Affiliations:** Department of Medical Oncology, Lu'an Hospital Affiliated to Anhui University of Chinese Medicine, China

## Abstract

**Background:**

The competing endogenous RNA (CeRNA) network plays important roles in the occurrence and development of colon cancer. This research is aimed at constructing a miRNA-mRNA network associated with exosomes in colon cancer.

**Methods:**

We explored the GEO database and then analyzed the RNAs of 722 samples to obtain differentially expressed miRNAs (DEMs) and mRNAs (DEGs) alongside the progress of colon cancer. Next, Gene Ontology (GO) and Kyoto Encyclopedia of Genes and Genomes (KEGG) analysis of DEM target genes and DEGs were performed. In addition, a miRNA-mRNA network related to exosomes in colon cancer was constructed based on DEMs and DEGs. Finally, the expression of miRNA and mRNA in the network was verified by GEPIA2 on the base of TCGA database.

**Results:**

Through our analysis, 19 DEMs (17 up and 2 down) and 1672 DEGs (954 up and 718 down) were screened. The GO and KEGG results show that these DEGs were mainly enriched in ribonucleoprotein complex biogenesis, noncoding RNA metabolic process, cell-substrate junction, cadherin binding, transcription coregulator activity, and regulation of the human T-cell leukemia virus 1 infection-related pathway. Besides, a miRNA-mRNA network, including 4 miRNAs (hsa-miR-623, hsa-miR-320c, hsa-miR-486-5p, and hsa-miR-1290) and 7 mRNAs (GNAI1, CADM1, PGRMC2, etc.), was constructed. Three of these seven mRNAs were downregulated in colon cancer. Ultimately, the GNAI1, CADM1, and PGRMC2 expression levels were verified by TCGA database.

**Conclusions:**

This study reveals the network relationship between colon cancer exosome-derived miRNA and targeted mRNA. It deepens our understanding of new molecular mechanisms and pathways that may play a role in the occurrence and metastasis of colon cancer.

## 1. Introduction

Colon cancer is one of the most frequent malignant tumors of the digestive system, and its global incidence is as high as 10.2%, second only to lung cancer and breast cancer [1]. Previous studies have shown that the risk of colon cancer increased with age, but in recent years, it has been found that the incidence of colon cancer in patients over 50 years old has decreased and the incidence of early-onset colon cancer has significantly increased. The incidence of colon cancer in patients aged 20–34 in the United States is expected to increase by more than 90% compared with 2010 by 2030 [2–4]. The mortality rate of colon cancer is affected by many factors, such as different countries, different dietary habits, and the timing of cancer screening programs [5], among which the disease stage is the major determinant. Therefore, it is especially important to detect paracancerous infiltration and related mechanisms early to reduce the risk of colon cancer metastasis [6, 7].

In recent years, numerous studies have documented that the risk of early local invasion and distant metastasis of colon cancer is related to exosomes secreted by primary tumor cells [8]. Most scholars believe that the formation process of exosomes is the endocytosis of the cell membrane to form endosomes, which are further fused into multivesicular bodies (MVB), and MVB forms vesicles through membrane invagination [9, 10]. The intraluminal vesicles (ILV) load a variety of substances (such as miRNA and mRNA) into it. After MVB fuses with the cell membrane, ILV is released outside of the cell by exocytosis through the cell membrane, forming extracellular vesicles of about 30–200 nm in size [11–13] that are exosomes. Exosomes mainly bind to the corresponding receptors on the surrounding or distant cell membranes through autocrine and paracrine [14, 15], thereby playing the role of material transmission and signal transduction. In the development of colon cancer, tumor-derived exosomes can mediate this process through a variety of ways, such as targeting thioesterase superfamily member 4- (THEM4-) mediated PI3K/AKT and NF-*κ*B pathways [16], targeting programmed cell death protein 4 (PDCD4) [17]. Teng et al. found that exosomes derived from primary colon tumors in mice promote oncogenesis and tumor progression of colon cancer by interacting with the major vault protein (MVP) [18]. Ma et al. experimentally confirmed that M2 macrophage-derived exosomes can target the ZC3H12B protein of the ZC3H12 protein family by transferring miR-155-5p to tumor cells and participate in the upregulation of IL-6 expression, thereby promoting the immune escape of colon cancer and accelerating tumor progression [19]. Bigagli et al. found through in vitro experiments that the exosomes secreted by human colon cancer cells affect the adhesion of adjacent tumor metastatic cells by regulating epithelial-mesenchymal transition, thereby preserving the microenvironment for tumor growth and ultimately promoting the metastasis of colon cancer [20]. However, it is not clear how the expression of miRNA targets and related characteristics in the exosomes secreted by colon cancer cells regulates the process of metastasis.

In eukaryotes, microRNA (miRNA) is a class of noncoding single-stranded small RNA with a length of 19–25 nucleotides, which is formed by the transformation of 300 to 1000 bases of endogenous primitive RNA into the cytoplasm through the process of intranuclear processing and digestion by enzymes and the transfer of exportin-5 (XPO5) [21]. The miRNA in the cytoplasm is loaded by Argonaute paralog 2 (AGO2) into the RNA-induced silencing complex (RISC), which plays an important role in regulating expression and silencing after complementary pairing with the base of the target mRNA [22, 23]. The information regulatory network composed of miRNA-mRNA is involved in the development of many diseases. For example, miR_21 promotes the growth of hepatocellular carcinoma by regulating the expression of tumor suppressor gene PTEN, PTENp1 gene [24], and miR_665 promotes breast cancer invasion and metastasis by activating the MAPK/ERK kinase (MEK) signal pathway [25]. Exosomes with low levels of miR-34c-3p accelerate the metastasis of non-small cell lung cancer by upregulating the level of synthase *α* 2 *β* 1 [26]. miR-182 is highly expressed in exosomes derived from gallbladder cancer, which can increase the expression level of N-cadherin and MMP2 by targeted inhibition of reversion-inducing-cysteine-rich protein with kazal motifs (RECK) and, finally, significantly promote the metastasis and invasion of gallbladder cancer cells [27]. Song et al. found that miR-9-5p was overexpressed in the exosomes derived from renal cancer and miR-9-5p targeted to the complementary sequence of the suppressor of cytokine signaling 4/5 mRNA (SOCS4/5), thus inhibiting translation and finally promoting the proliferation and invasion of human renal cancer cell line A-704 [28].

**Figure 1:**
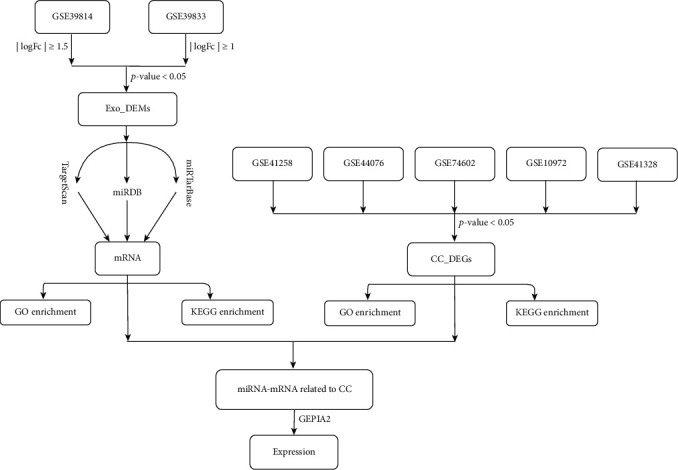
Workflow of this study. Analyzed the RNAs to obtain DEMs and DEGs alongside the progress of colon cancer. GO and KEGG analyses were then performed on the DEM target genes and DEGs. Ultimately, a miRNA-mRNA network associated with colon cancer exosomes was constructed.

Previous studies have shown that circulating miRNAs from sources such as blood are mainly involved in the development of colon cancer by regulating target genes and acting as oncogene or tumor suppressor gene. In contrast, it is not clear how the exosomes derived from colon cancer tissues, which carry miRNAs, regulate the whole disease process of colon cancer. In this paper, we analyzed the miRNAs of colon cancer exosomes and their corresponding target genes. We further investigated the potential signaling regulatory pathways of DEM target genes, as well as those of DEGs. Finally, a miRNA-mRNA interaction network was constructed. Through our study, we elucidated the possible interactions and regulatory relationships between exosome-derived miRNAs and colon cancer, throughout the developmental process. This provides new ideas for our understanding of colon cancer as a disease and its subsequent diagnosis and treatment. The workflow of this study is illustrated in Figure 1.

## 2. Materials & Methods

### 2.1. Data Download

Firstly, we searched the GEO database (Gene Expression Omnibus (https://www.ncbi.nlm.nih.gov/geoprofiles) for datasets related to colon cancer and exosomes by using the keywords “colon cancer” and “exosomes.” Finally, seven GEO datasets were obtained that met the requirements. We summarized the data sources and attributes of the datasets (Table 1). For miRNA expression profiling related to exosomes in colon cancer, 6 exosome samples from colon cancer cell lines and 3 exosome samples from normal colon-derived epithelial cells were enrolled in GSE39814, while exosomal miRNAs in sera of colon cancer patients (*n* = 88) and healthy controls (*n* = 11) were enrolled in GSE39833.

Then, for gene/mRNA expression profiling in colon cancer, we included the datasets GSE41258, GSE44076, GSE74602, GSE10972, and GSE41328. These datasets contain colon cancer tissue samples and the corresponding normal tissues, respectively.

### 2.2. DEM and DEG Screening

Firstly, the differentially expressed miRNAs between exosome samples from colon cancer and matched normal colon-derived epithelial cells screened by GEO2R online software [29] (https://www.ncbi.nlm.nih.gov/geo/geo2r/), which is dependent on the R programming “LIMMA” package, were utilized to recognize DEMs and DEGs between colon cancer samples and normal examples [30]. During the period, the expression data was normalized by “normalizeBetweenArrays” function in “limma” package from R software [31]. For GSE39814, a *P* value < 0.05 and |logFC| ≥ 1.5 were selected as the threshold. For GSE39833, a *P* value < 0.05 and |logFC| ≥ 1 were considered as the threshold. At the same time, the differentially expressed mRNAs in colon cancer samples and normal colonic tissues were also screened by GEO2R online software. These differentially expressed mRNAs with a *P* value < 0.05 were selected for further analysis. Next, the overlapped differentially expressed miRNAs in GSE39814 and GSE39833 were selected as DEMs; the overlapped differentially miRNAs expressed in GSE41258, GSE44076, GSE74602, GSE10972, and GSE41328 were selected as DEGs. The Venn diagrams of all overlapping DEMs and DEGs were created using the website http://bioinformatics.psb.ugent.be/webtools/Venn/.

### 2.3. GO and KEGG Enrichment Analysis of DEM Target Genes and DEGs

In order to forecast the possible functions and sum up a more overall signal pathway of many DEM target genes and DEGs that look messy and to further judge the significance of the genes of concern, the Kyoto Encyclopedia of Genes and Genomes (KEGG) pathway and Gene ontology (GO) analysis were conducted by the *clusterProfiler* R package [32]. In this study, GO analysis was conducted from three distinguished aspects: biological process (BP), cellular component (CC), and molecular function (MF). *P* value < 0.05 was considered statistically significant.

### 2.4. miRNA-mRNA Network Construction

As we know, miRNA can bind to targeted mRNA to promote the degradation of mRNA. Herein, target genes of these miRNA signatures were obtained by using miRDB, miRTarBase, and TargetScan databases. Genes present in all three databases were regarded as target genes of these miRNAs. Comparing predicted target genes with DEGs, only the remaining overlapped genes and their interaction pairs were used for constructing the miRNA-mRNA pairs. Therefore, the miRNA-mRNA network was related to exosomes in colon cancer.

### 2.5. Validation of the miRNA-mRNA Network

The expression levels of miRNA in the miRNA-mRNA network were verified by CancerMIRNome (http://bioinfo.jialab-ucr.org/CancerMIRNome) on the base of the TCGA database. The expression levels of mRNA in the miRNA-mRNA network were verified by Gene Expression Profiling Interactive Analysis (GEPIA2; http://gepia2.cancer-pku.cn/index) on the base of the TCGA database.

## 3. Results

### 3.1. DEM and DEG Screening

**Figure 2:**
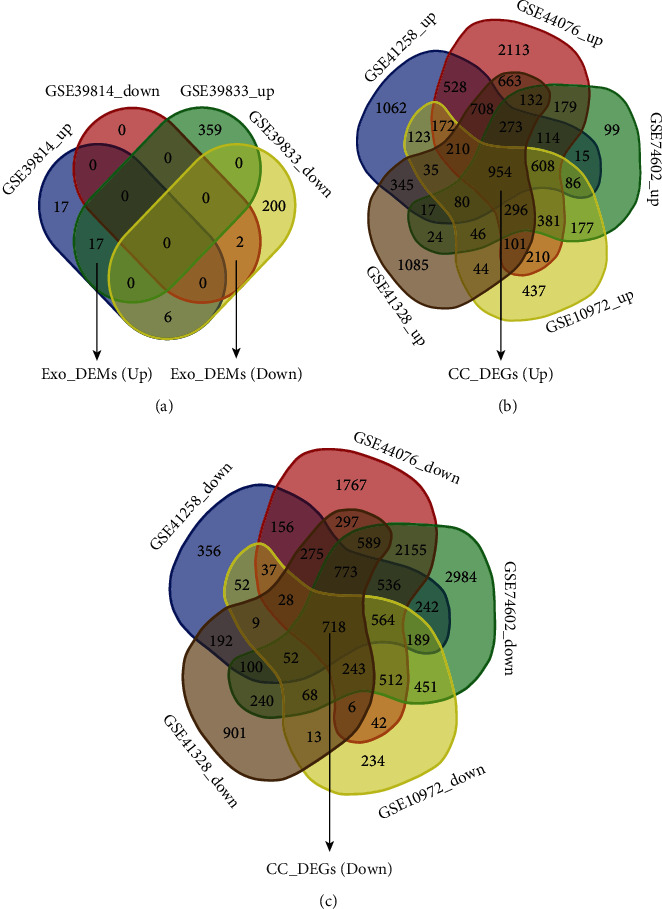
Venn of DEMs and DEGs. (a) Exo_DEMs screening. Datasets GSE39814 and GSE39833 were screened by Venn diagrams for 17 upregulated EXO-DEMs and 2 downregulated EXO-DEMs, respectively. (b, c) DEGs of colon cancer screening. The datasets GSE41258, GSE44076, GSE74602, GSE10972, and GSE41328 were screened by the Venn diagram to identify 954 upregulated DEGs and 718 downregulated DEGs in colon cancer patients.

**Figure 3:**
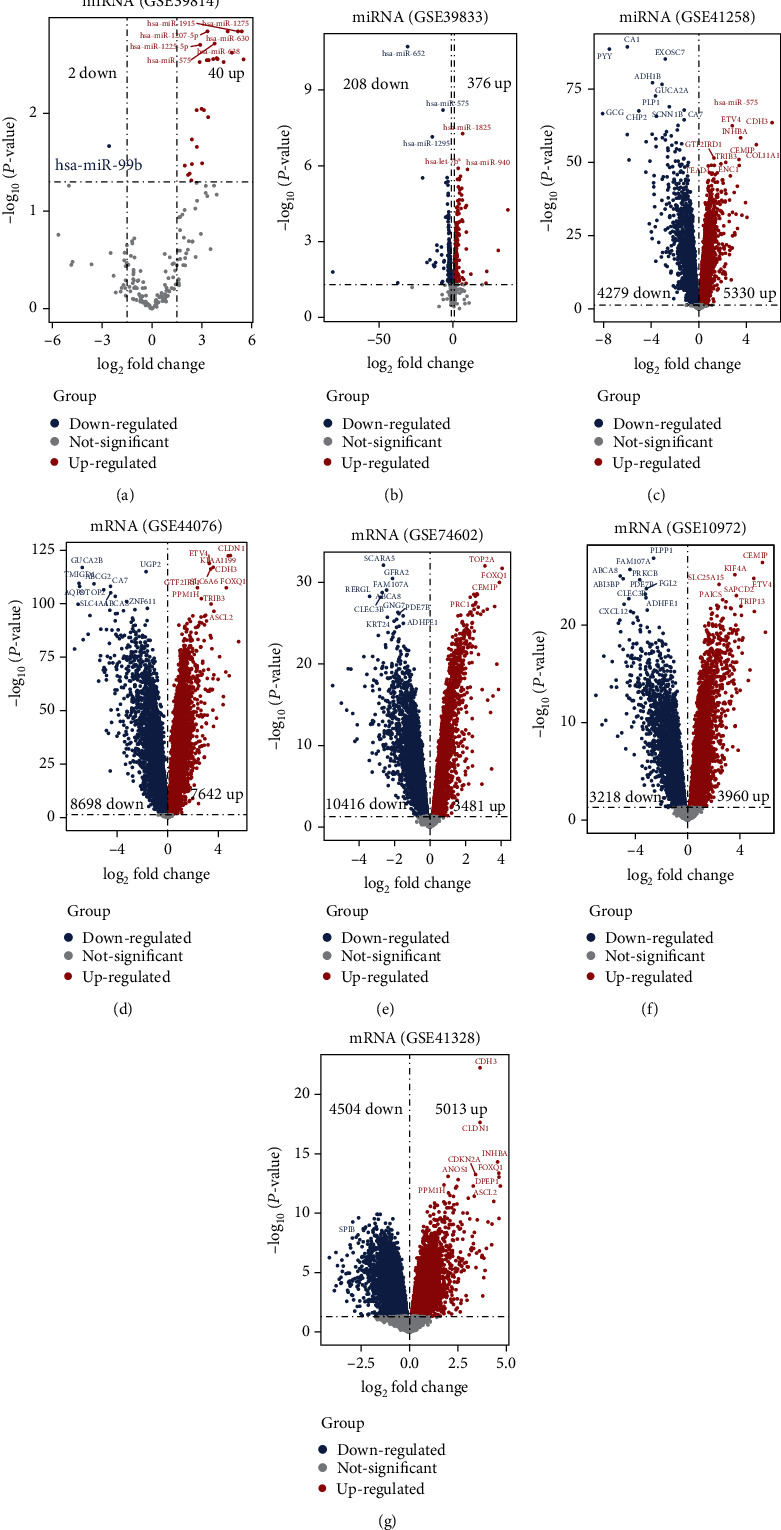
Volcano plot of DEMs and DEGs. Volcano map of the differentially expressed miRNAs (DEMs) and genes (DEGs). Blue spots represent downregulated expression, gray spots represent nonsignificant expression, and red spots represent upregulated expression. (a, b) The distribution of DEMs between exosome samples from colon cancer and matched normal colon-derived epithelial cell; the samples come from the datasets GSE39814 and GSE39833 (DEMs of GSE39814 were selected with thresholds of fold change > 1.5 and *P* < 0.05; DEMs of GSE39833 were selected with thresholds of fold change > 1 and *P* < 0.05); (c–g) the distribution of DEGs between colon cancer samples and normal colonic tissues; the samples come from the datasets GSE41258, GSE44076, GSE74602, GSE10972, and GSE41328 (these DEGs with a *P* value < 0.05 were selected).

Data were separately analyzed (Table 1). 19 DEMs (17 up and 2 down) and 1672 DEGs (954 up and 718 down) were screened (Figure 2). As for DEMs (Figure 3), there were 42 differentially expressed miRNAs (40 up and 2 down) in GSE39814, while there were 584 differentially expressed miRNAs (376 up and 208 down) in GSE39833. 19 overlapped miRNAs were screened. As for DEGs, 9609 were differentially expressed mRNAs (5330 up and 4279 down) in GSE41258, 16340 were differentially expressed mRNAs (7642 up and 8698 down) in GSE44076, 13897 were differentially expressed mRNAs (3481 up and 10416 down) in GSE74602, 6908 were differentially expressed mRNAs (3960 up and 3218 down) in GSE10972, and 9517 were differentially expressed mRNAs (5013 up and 4504 down) in GSE41328. 1672 overlapped mRNAs were screened. Next, the DEMs and DEGs were used to the next analysis.

### 3.2. GO and KEGG Enrichment Analysis of DEM Target Genes and DEGs

**Figure 4:**
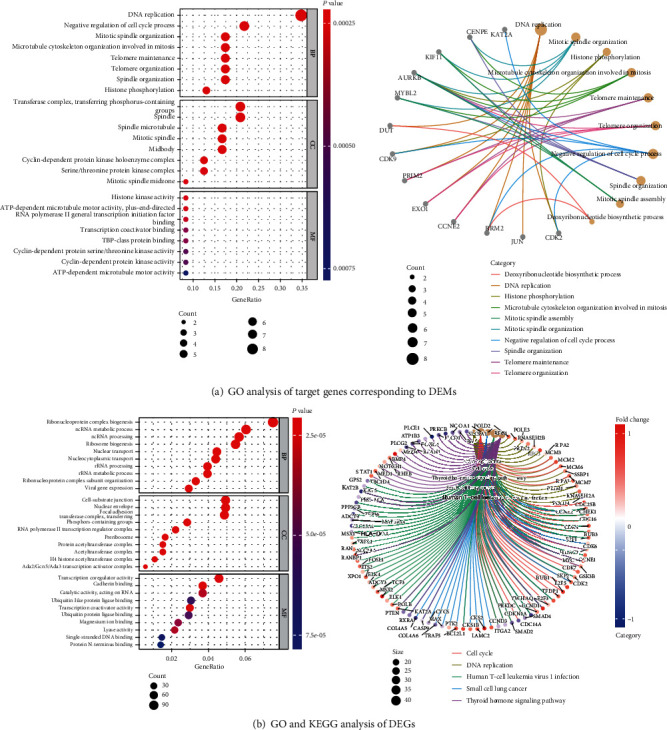
GO and KEGG analysis. GO and KEGG pathway enrichment analysis of DEM target genes and DEGs. (a) GO analysis of target genes corresponding to DEMs. Biological process (BP), cellular component (CC), and molecular function (MF). (b) GO and KEGG analysis of DEGs. KEGG pathway enrichment analysis based on identified DEGs (a *P* value < 0.05 was considered statistically significant).

As depicted in Figure 4(a), GO describes DEM target genes in terms of biological processes (BP), cellular components (CC), and molecular functions (MF). In the BP group, the differential genes were mainly enriched in cell cycle regulatory processes such as “DNA replication” and “negative regulation of cell cycle process.” In the CC group, the differential genes were mainly enriched in “transferase complex, transferring phosphorus-containing” and other mitotic spindle and enzyme components. In the MF group, the differential genes were mainly enriched in enzymes and molecular activities such as “histone kinase activity,” as well as binding and movement of various factors.

As shown in Figure 4(b), in the BP group, the differential genes were mainly enriched in “ribonucleoprotein complex biogenesis,” “ncRNA metabolic process,” “ncRNA processing,” “ribosome biogenesis,” “nuclear transport,” and so on. In the CC group, the differential genes were mainly based on “cell-substrate junction,” “nuclear envelope,” “focal adhesion,” “transferase complex, transferring,” “phosphorus-containing groups,” and so on. In the MF group, the differential genes were mainly based on “transcription coregulator activity,” “cadherin binding,” “catalytic activity, acting on RNA,” “transcription coactivator activity,” “lyase activity.” In KEGG pathway enrichment analysis, the differential genes are mainly based on “infection,” “material metabolism,” “cell cycle,” “signal pathway,” and “tumor.”

Combined with GO and KEGG enrichment results, we compared and further analyzed that there are relatively many studies on DNA replication and cell cycle, such as CDK4, MYC, and MCM. However, in the GO enrichment analysis of differential genes in colon cancer, we also enriched some genes that are less studied or even never studied in colon cancer, such as POLD2, CDC16, PRIM2, etc. At the same time, some of the pathways of KEGG enrichment are relatively rare in colon cancer, such as the AGE-RAGE signal pathway in diabetic complications, oxytocin signal pathway, and coronavirus disease COVID-19. Whether these less studied pathways and genes play an important role in the pathogenesis of colon cancer remains to be further investigated.

### 3.3. miRNA-mRNA Network

**Figure 5:**
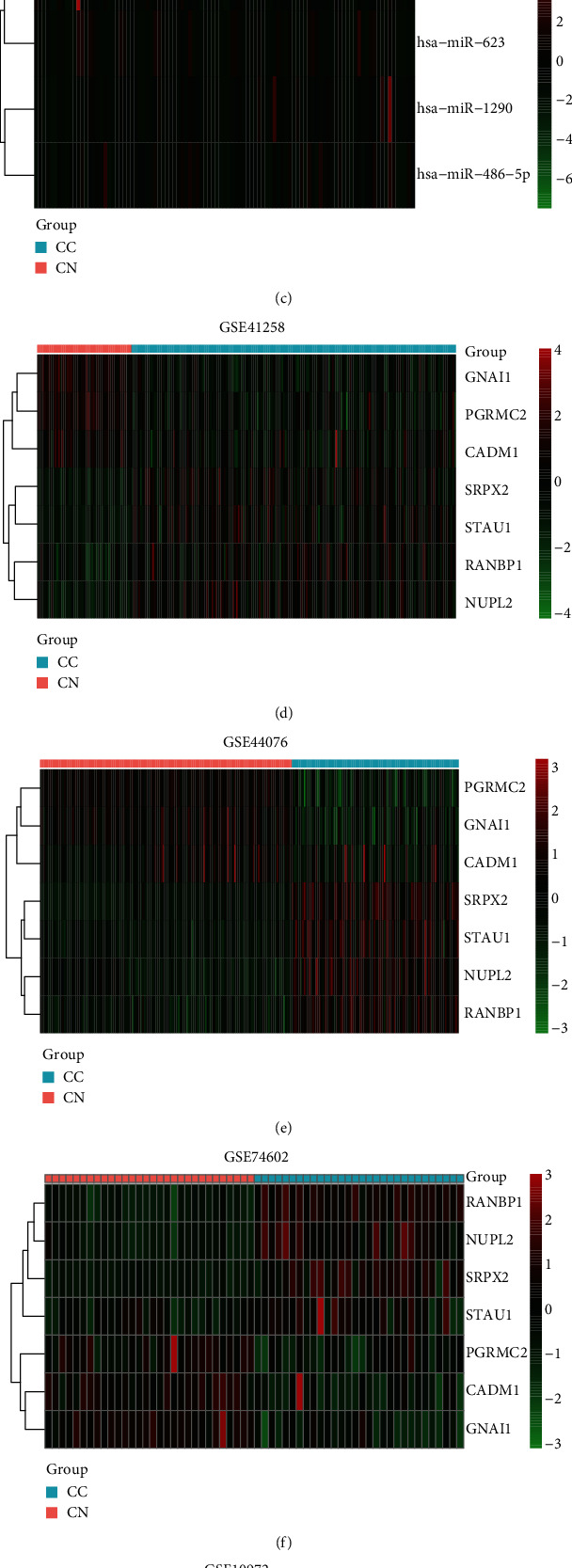
miRNA-mRNA network and heatmap. The color bar above the heat map represents the sample group, light-red represents normal samples, and light-blue represents tumor samples. (a) miRNA-mRNA network. The triangle represents the miRNA, and the ellipse represents mRNA. The red color refers to an upregulation, while the green color refers to a downregulation. 4 miRNAs were upregulated and 3 mRNAs were downregulated in colon cancer. (b, c) Heatmap of miRNA in the miRNA-mRNA network. Heat map of hierarchical clustering of colon cancer samples based on the 4 differentially expressed miRNAs. (d–h) Heatmap of mRNA in the miRNA-mRNA network. Heat map of hierarchical clustering of colon cancer samples based on the 7 differentially expressed mRNAs.

**Figure 6:**
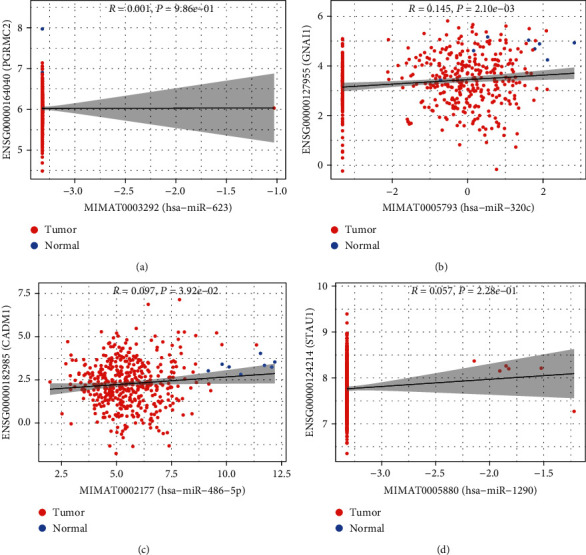
Pearson correlation analysis of the miRNA and its target genes. The red dots are tumor samples and the blue dots are normal samples. Statistical significance was defined as ^∗^*P* < 0.05. *R* > 0.2 was defined as miRNA, and target genes were correlated between them.

As illustrated in Figure 5, a miRNA-mRNA network, including 4 miRNAs (hsa-miR-623, hsa-miR-320c, hsa-miR-486-5p, and hsa-miR-1290) and 7 mRNAs (GNAI1, CADM1, PGRMC2 etc.), was constructed. Those 4 miRNAs were upregulated and 3 mRNAs were downregulated in colon cancer. Of those, the expressions of hsa-miR-623, hsa-miR-320c, and hsa-miR-486-5p were upregulated while their corresponding target genes PGRMC2, GNAI1, and CADM1 were downregulated. We further analyzed the Pearson correlation analysis of these 4 differential miRNAs and their corresponding target genes, as shown in Figure 6.

### 3.4. Validation of the miRNAs in the miRNA-mRNA Network

**Figure 7:**
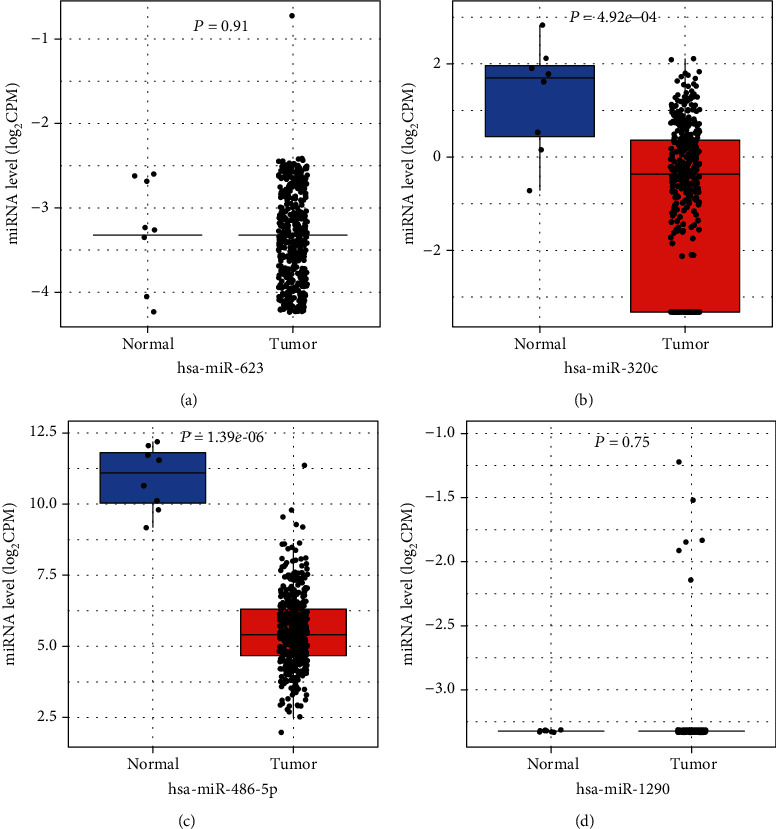
The expression level of miRNA in the miRNA-mRNA network. The expression levels of hsa-miR-623, hsa-miR-320c, hsa-miR-486-5p, and hsa-miR-1290 in colon cancer and normal sample based on the TCGA database analyzed by CancerMIRNome (http://bioinfo.jialab-ucr.org/CancerMIRNome). The red plot represents the tumor sample, and the blue plot represents the normal sample. ^∗^*P* < 0.05 was considered statistically significant. The expression levels of hsa-miR-320c and hsa-miR-486-5p in colon cancer and normal specimens were statistically different.

As showcased in Figure 7, the CancerMIRNome database (http://bioinfo.jialab-ucr.org/CancerMIRNome) was used to verify the expression levels of the 4 miRNAs in colon cancer samples and normal tissues. The expression levels of hsa-miR-320c and hsa-miR-486-5p in colon cancer and normal specimens were statistically different.

**Figure 8:**
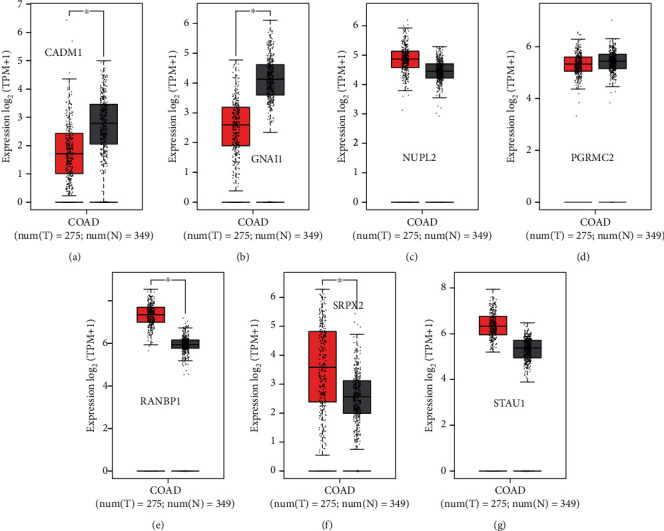
The expression level of mRNA in the miRNA-mRNA network. The expression levels of CADM1, GNAI1, NUPL2, PGRMC2, RANBP1, SRPX2, and STAU1 in colon cancer and normal sample based on the TCGA database analyzed by GEPIA. The red plot represents the tumor sample, and the grey plot represents the normal sample. ^∗^*P* < 0.05 was considered statistically significant. The expression levels of CADM1, GNAI1, and PGRMC2 in colon cancer and normal specimens were statistically different. CADM1: cell adhesion molecule 1; GNAI1: G protein subunit alpha i1; NUPL2: nucleoporin like 2; PGRMC2: progesterone receptor membrane component 2; RANBP1: RAN-binding protein 1; SRPX2: sushi repeat containing protein X-linked 2; STAU1: staufen double-stranded RNA-binding protein 1.

As showcased in Figure 8, the GEPIA2 database was used to verify the expression levels of the 7 mRNAs in colon cancer samples and normal tissues. The expression levels of the 3 mRNAs were downregulated, and 4 mRNAs were upregulated in colon cancer samples on the basis of gene expression profiles from The Cancer Genome Atlas (TCGA) and the genotype-tissue expression (GTEx) projects. The results of these 7 mRNA expression trends in colon cancer patients and healthy people based on the GEPIA2 database were in accordance with those based on the GEO datasets.

## 4. Discussion

Although colon cancer conditions had a high prevalence, about 39% of new patients in the United States are diagnosed with localized lesions that can be operated on [7]. Therefore, how to detect and diagnose colon cancer early and accurately is an urgent problem to get a better prognosis and a longer survival time. With the continual advancements in biomedicine, gene therapy has attracted more and more attention as a precise, efficient, and new cutting-edge technology for colon cancer. Researchers have achieved efficacy in a series of in vitro and in vivo studies through gene regulation [38, 39] and delivery system improvement [40, 41]. Therefore, how to accurately identify colon cancer-related genes has become the key to effective treatment. Studies have shown that many types of cells, including stem cells, nerve cells, and tumor cells, can release exosomes. Exosomes contain large amounts of nucleic acids and their metabolites, proteins, and lipids [42], while colon cancer exosome-derived miRNA exchange information with normal cells by them to promote tumor angiogenesis and induce tumor metastasis [43]. Our study is aimed at finding out the interaction of the miRNA-mRNA network in the progression of colon cancer, thus providing new ideas and methods for the early diagnosis and treatment of colon cancer.

To further clarify the interrelationship between miRNAs in colon cancer-derived exosomes and the process of colon cancer development and progression, we performed a comprehensive analysis of exosome-derived miRNAs and gene expression profiles of colon cancer in the GEO database.19 DEMs (17 upregulated and 2 downregulated) and 1672 DEGs (954 upregulated and 718 downregulated) were isolated. GO and KEGG enrichment analysis of these DEGs was carried out. At the same time, the DEMs in the exosome were conservatively predicted by multiple databases after the intersection of DEMs and the predicted mRNA was intersected with DEGs again to form a final miRNA-mRNA network, which consisted of 4 miRNAs and 7 mRNAs (4 upregulated and 3 downregulated). The expression trend of these 7 mRNAs based on the GEPIA2 database in colon cancer patients and healthy people was consistent with the results that we obtained. As miRNAs that intersect with DEGs of colon cancer patients, its data come from two datasets GSE39814 and GSE3988. Among them, GSE39814 uses the exosomes of human colonic adenocarcinoma epithelial cells (HCT116 and SW480), compared with the exosomes of normal colonic epithelial cells. GSE39833 uses tumor sample exosomes from colon cancer patients with different TNM stages to compare the peripheral serum exosomes of patients after colon cancer surgery. As the data source of DEGs, GSE41258 and other datasets took colon cancer tissues compared with the surrounding normal tissues. In this screening process, the miRNA carried by exosomes not only originated from different cell lines but also encompasses samples from colon cancer patients with different TNM stages but the final predicted DEMs and DEGs were involved in the occurrence and development of various types of tumors, which also reinforces the biological significance of the research results. The deficiency is that it is just a process of data analysis and the association between its target gene and colon cancer needs to be confirmed by more data. However, it is precisely due to the lack of exocrine data sources of primary colon cancer and the relationship between miRNA and colorectal oncogenes and signal pathways remains elusive, which is precisely our potential research direction in the future.

The 4 obtained miRNAs were hsa-miR-623, hsa-miR-320 C, hsa-miR-486-5P, and hsa-miR-1290, and 7 mRNAs were NUPL2, RANBP 1, SRPX2, STAU1, PGRMC2, CADM1, and GNAI1. It is worth mentioning that the differentially expressed exosomal miRNAs that we predicted were mainly involved in cGMP-PKG and chemokine signaling pathways. As the second messenger of intracellular information transmission, the cyclic guanosine monophosphate- (cGMP-) related signal pathway plays an important role in the treatment of colon [44]; the cGMP-PKG dimer and related pathways formed by cGMP-dependent protein kinase G (PKG) enhance the role of the original cGMP-monomer as a second messenger for intracellular communication, which plays an important role in inhibiting the progression of colon cancer [45, 46]. The chemokine family of small molecular and protein components can stimulate migration in various cell types, and chemokines are involved in many kinds of tumor progression [47, 48]. These data show that the screened exosome mi-RNA is reliable and may affect tumor genesis and development. Then, we further completed the Pearson correlation analysis of DEMs with target genes. Although the correlation was not very statistically significant, we observed that it was a result of the small sample size in the normal group, but overall, it also reflected some degree of variation in the trend.

Then, we found that in previous cancer studies, the miRNAs carried by the four key exosomes that we identified have all been reported but they were poorly studied in colon cancer and some of them had not even been carried out. Studies have shown that the role of hsa-miR-623 as a tumor suppressor has been confirmed, such as targeting the effect of XRCC5 to inhibit the proliferation and metastasis of cancer cells such as breast cancer and liver cancer [49], inhibit the proliferation of gastric cancer cells, and increase the drug sensitivity of 5-fluorouracil by targeting cyclin D1 [50]. At the same time, hsa-miR-623 may be used as a predictor of the poor prognosis of breast cancer [51]. What attracts our particular attention is that the interaction between hsa-miR-623 and colon cancer has never been reported and the exosomes, as the source of hsa-miR-623, have never been studied. hsa-miR-320c is a biomarker for early detection in some studies, for example, it has a high detection rate in early-stage esophageal squamous cell carcinoma [52]. In terms of colon cancer, there is a significant correlation between miR-320c levels in plasma exosomes and nerve invasion [53]. Wang et al. found that exosomes of colon cancer cells promoted the progression of colon cancer by inhibiting the expression level of interferon regulatory factor 4 (IRF4) in regulatory T cells (Tregs) by transferring miR-320c [54]. Overall, there is a paucity of studies on miR-320c and further in vitro and in vivo studies are needed to confirm its association with colon cancer. hsa-miR-486-5p has been confirmed as a high-quality biomarker for the diagnosis of non-small cell lung cancer [55, 56]. In terms of colon cancer, Kelley et al. found that it is differentially expressed in early and advanced colon cancer samples and its downregulation may indicate the occurrence of primary colon cancer [57]. Ye et al. found that hsa-miR-1290 was positively correlated with the state of different mismatch repair (dMMR). By inhibiting the expression of hsa-miR-1290, the sensitivity of the samples to 5-fluorouracil was improved [58]. Ma et al. demonstrated that miR-1290 targeting inositol polyphosphate 4-phosphatase B (INPP4B) induces proliferation of colorectal cancer cells [59].

The exosome secreted by tumor cells may regulate tumor progression by releasing the information that it carries [60], in which the miRNA-mRNA network plays an important role. Our enrichment analysis of DEM target genes and DEGs revealed that many of them are associated with molecules and pathways in tumor metabolism and cell cycle. We further analyzed the seven mRNA associated with the four key miRNA and learned that NUPL2 in the four upregulated genes predicted response to radiation and chemotherapy in patients with locally advanced rectal cancer [61]. RANBP1 inhibits the proliferation of colorectal tumors [62], which negatively correlated with the paclitaxel sensitivity of colorectal cancer cells [63]. SRPX2 promotes glycolysis in colon cancer cells [64]. STAU1 influences colon cancer expression by regulating RNA subset localization during mitosis [65]. We speculate that among the three downregulated genes more closely related to colon cancer, PGRMC2 is associated with the progression of a variety of tumors [66, 67] but no related reports have been found in colon cancer. Tsuboi et al. found that CADM1 could interfere with carcinogenic signal transduction and inhibit the occurrence of colon cancer by binding to Csk-binding protein (Cbp) [68]. More studies suggest that CADM1 is involved in the occurrence of cervical cancer [69], breast cancer [70], and liver cancer. Li et al. found that GNAI1 inhibits the occurrence of colon cancer by blocking signal transduction and downregulating the level of GNAI2 [71].

Based on the abovementioned literature and analysis, we speculate that exosomes secreted by colon cancer tissue have multiple regulatory axes in mediating the tumorigenesis and metastasis of colon cancer. In our predicted miRNA-mRNA network, some miRNAs, such as hsa-miR-320 c, are poorly studied in colon cancer. In addition, what is more worthy of our attention is that some miRNAs have not been reported in colon cancer according to the data that we have, such as hsa-miR-623. As the source of hsa-miR-623, the interaction between exosomes and colon cancer is not clear. Therefore, we predicted that the pathways composed of highly expressed miRNA and downregulated target genes, such as hsa-miR-320c/GNAI1, hsa-miR-623/PGRMC2, and hsa-miR-486-5p/CADM1 are worthy of further exploration.

## 5. Conclusions

We have constructed a potential miRNA-mRNA regulatory network, and several candidate targets were identified to be effectively (hsa-miR-623, has-miR-320C, has-miR-486-5p, has-miR-1290, NUPL2, RANBP1, SRPX2, STAU1, PGRMC2, CADM1, and GNAI1) involved in the pathogenesis of colon cancer, which provides a new direction and potential therapeutic targets for colon cancer research. In the future, we may improve the therapeutic effect and prognosis of colon cancer patients by targeting the existing miRNA-mRNA network but more data needed for derivation and further verification in vivo and in vitro experiments.

## Figures and Tables

**Table 1:** Data sources and attributes.

GEO accession	Sample size		Platform	Experiment type
	Normal samples	CC samples		
GSE39814 [33]	3	6	Agilent-021827 Human miRNA Microarray G4470C (feature number version)	Noncoding RNA profiling by array
GSE39833 [33]	11	88	Agilent-021827 Human miRNA Microarray G4470C (feature number version)	Noncoding RNA profiling by array
GSE41258 [34]	54	186	[HG-U133A] Affymetrix Human Genome U133A Array	Expression profiling by array
GSE44076 [35]	148	98	[HG-U219] Affymetrix Human Genome U219 Array	Expression profiling by array
GSE74602	30	30	Illumina humanRef-8 v2.0 expression beadchip	Expression profiling by array
GSE10972 [36]	24	24	Illumina humanRef-8 v2.0 expression beadchip	Expression profiling by array
GSE41328 [37]	10	10	[HG-U133_Plus_2] Affymetrix Human Genome U133 Plus 2.0 Array	Expression profiling by array

CC: colon cancer.

## Data Availability

The datasets for the relevant analyses in the manuscript can be obtained by entering the corresponding dataset numbers in the GEO database.
